# Event trigger based adaptive neural trajectory tracking finite time control for underactuated unmanned marine surface vessels with asymmetric input saturation

**DOI:** 10.1038/s41598-023-37331-6

**Published:** 2023-06-22

**Authors:** Yancai Hu, Qiang Zhang, Yang Liu, Xiangfei Meng

**Affiliations:** 1 Navigation College, Shandong Jiaotong University, Jinan, China; 2 Division of Maritime Transportation, Mokpo National Maritime University, Mokpo-Si, Korea; 3 Merchant Marine College, Shanghai Maritime University, Shanghai, China

**Keywords:** Computational science, Applied mathematics, Mechanical engineering

## Abstract

An adaptive finite time trajectory tracking control method is presented for underactuated unmanned marine surface vessels (MSVs) by employing neural networks to approximate system uncertainties. The proposed algorithm is developed by combining event-triggered control (ETC) and finite-time convergence (FTC) techniques. The dynamic event-triggered condition is adopted to avert the frequent acting of actuators using an adjustable triggered variable to regulate the minimal inter-event times. While solving the system uncertainties and asymmetric input saturation, an adaptive neural networks based backstepping controller is designed based on FTC under bounded disturbances. In addition, via Lyapunov approach it is proved that all signals in the closed-loop system are semi-global uniformly ultimately bounded. Finally, simulations results are shown to demonstrate the effectiveness of this proposed scheme.

## Introduction

Ships sailing under complex sea conditions have great dynamic uncertainty. The unknown time-varying interference and the limited communication resources affect the trajectory tracking of underactuated surface ships. There are many urgent problems in the trajectory tracking control and actual navigation engineering. An effective control scheme is very important for MSVs^1–5^, which is conducive to improving the tracking accuracy of ships and enabling them to complete the tracking task safely and efficiently. To make the control scheme easier to implement in engineering practice, many practical factors need to be considered, such as input saturation, state constraints, communication resource constraints, etc.

The MSV trajectory tracking system is a classical nonlinear control system. The increased efforts have been taken to research the motion control of this system in the past decades. Control laws are designed for MSV to track the time referenced trajectory or virtual objects^6^. It is significant to apply trajectory tracking in scenarios of way-point navigation, reconnaissance and surveillance. Attentions have been paid to trajectory tracking control both in theory and practice^7–9^. The paper^10^ studied the robust tracking control of underactuated surface vessels with parameters uncertainties using sliding mode control technique. The paper^11^ constructed an observer to estimate unknown disturbances and design a robust trajectory tracking controller through backstepping method for MSVs. Lekkas^12^ applied a tracking guidance system for MSVs considering unknown ocean currents. Moreover, linear algebra method^13^, sampled-data approach^14^ and model predictive nonlinear control^15^ have been adopted to design the trajectory tracking controller for MSVs.

When designing the trajectory tracking controller, difficulties caused by external disturbances and dynamic uncertainty need to be conquered. Thus, neural networks^16–18^, fuzzy logic^19–23^, sliding mode^24^ and adaptive backstepping^25–27^ have been continuously integrated and applied for the trajectory tracking control of MSVs. Adaptive control based on neural networks was used to approximate uncertainty and disturbance of nonlinear system^28,29^. The update laws of parameters were designed based on Lyapunov theorem.

The tracking ability of MSVs within limited time help to avoid collision with target ships and obstacles urgently and reasonably and improve the safety in complex environment. For realizing fast tracking control, finite time control of MSVs is designed to track desired trajectory within a limited time. The paper^30^ proposed a novel hyperbolic tangent guidance method to cooperatively control the course of the ship, and designed the controller using terminal non-singular sliding mode technology. The finite time control scheme greatly improves the convergence speed of the system. However, with the improvement of control accuracy, high energy consumption is often required and it also undoubtedly increase the wear and tear of the thrusters and controllers. In order to solve such problems, event-triggered technology has been applied. Tabuada^31^ first developed an event-triggered control scheme with static trigger conditions. The applications of event-triggered in ship motion and control have been further developed in papers^32,33^.

This paper presents a trajectory tracking control algorithm for unmanned MSVs with external disturbances considering saturation problem of actuators. The contributions of this paper have been summarized.

1. Gaussian error functions are introduced to explore saturation non-linearity which is represented by the continuous derivative formulation.
2. Based on finite time control, RBF neural networks is applied for the adaptive backstepping control method of MSVs with disturbances and actuator saturation.
3. Stability analysis is provided for the closed-loop systems. It proves all the states are semi-globally uniformly ultimately bounded. Tracking error converges to a small neighborhood of origin.

This paper is organized as follows. Section “Preliminaries and problem statements” states some useful preliminaries and problem formulation. Section “Controller design” is devoted to the algorithm of designing the trajectory tracking for MSVs. Simulations of the proposed control approach are introduced in Section “Stability proof”. Finally in Section “Simulation”, we make conclusions and propose further work.

## Preliminaries and problem statements

### Preliminaries

#### Notations

In this paper, $$\left| \cdot \right|$$ denotes the absolute value of a scalar or each component for a vector. For example, $${\text{x}} \in {\mathbb{R}}^{n}$$ is a vector, $$\left| x \right| = \left[ {\left| {x_{{1}} } \right|,\left| {x_{2} } \right|,....\left| {x_{n} } \right|} \right]^{T}$$. $$\left\| \cdot \right\|$$ denotes the Euclidean norm of a vector or the Frobenius norm of a matrix. $${\text{tr}}\left( X \right)$$ represents its trace with the property $${\text{tr}}\left( {X^{{\text{T}}} X} \right) = \left\| X \right\|^{2}$$ for a matrix $$X \in {\mathbb{R}}^{n \times n}$$ and $$a < b$$ represents $$a_{i} < b_{i} ,\;$$$$i = 1,2,...,n$$ for any vectors $$a \in {\mathbb{R}}^{n}$$ and $$b \in {\mathbb{R}}^{n}$$.

#### RBFNNs approximation

By using RBFNNs^34^, an unknown smooth nonlinear function $f\left( x \right)$, ${\mathbb{R}}^{m} \to {\mathbb{R}}$ can be approximated in a compact set $\Omega \subseteq {\mathbb{R}}^{m}$ as bellow

1 $$ f\left( x \right) = \omega^{*T} \cdot \Phi \left( x \right) + \Delta $$

where $\Delta$ is the approximation error that is bounded over $\Omega$, namely, $\Delta \le \overline{\Delta }$, $\overline{\Delta }$ is an unknown constant. $\omega^{*} \in {\mathbb{R}}^{l}$ represents the optimal weight vector, $l$ the node number of the NNs.

$\omega^{*} \in {\mathbb{R}}^{l}$ is defined as

2 $$ \omega^{*} = \arg \mathop {\min }\limits_{{\hat{\omega }}} \left\{ {\mathop {\sup }\limits_{x \in \Omega } \left| {f\left( x \right) - \hat{\omega }^{T} \Phi \left( x \right)} \right|} \right\} $$

where $\hat{\omega }$ is the estimation of $\omega^{*}$. $\Phi \left( x \right) = \left[ {\Phi_{{1}} \left( x \right),...,\Phi_{l} \left( x \right)} \right]^{T}$: $\Omega \to {\mathbb{R}}^{l}$ represents the RBF vector and elements are chosen as the Gaussian functions

3 $$ \Phi_{i} \left( x \right) = \exp \left( { - \frac{{\left\| {x - \mu } \right\|^{2} }}{{\varepsilon_{i}^{2} }}} \right){,}\;i = {1},...,l $$

where $\mu_{i} \in {\mathbb{R}}^{m}$ is the center and $\varepsilon_{i} \in {\mathbb{R}}^{m}$ is the spread.

#### Asymmetric inputs saturation

An auxiliary system is designed to describe the inputs saturation nonlinearity for backstepping method. This smooth auxiliary system with the asymmetric saturation nonlinearity is formulized as

4 $$ \begin{gathered} \tau_{i} \left( {\varphi_{i} } \right) = \tau_{Mi} + erf\left( {\frac{\sqrt \pi }{{2\tau_{Mi} }}\varphi_{i} } \right){,}\;i = {1,2,3} \hfill \\ \tau_{Mi} = \left( {\left( {\tau_{i}^{ + } + \tau_{i}^{ - } } \right)/2 + \left( {\tau_{i}^{ + } - \tau_{i}^{ - } } \right)/2} \right){\text{sign}} \left( {\varphi_{i} } \right){,}\; \hfill \\ \end{gathered} $$

where $\tau_{i}^{ + }$,$\tau_{i}^{ - }$ are the upper and lower bounds of the actuator,${\text{sign}} \left( {\varphi_{i} } \right)$ is the standard sign function, and $erf{(} \cdot {)}$ is a Gaussian error function with $erf{(}x{) = }\frac{2}{\sqrt \pi }\int_{{0}}^{x} {e^{{ - t^{2} }} } dt$. It shows the saturation limitation with smooth form in Fig. 2, with $\tau_{i}^{ + } = 5$,$\tau_{i}^{ - } = - 2.5$, and the input signal $\varphi \left( t \right) = 10{\text{sin}}\left( {2t} \right)$.

##### Remark 1

For the lower and upper bounds of $\tau_{i}^{ + }$,$\tau_{i}^{ - }$, if $\left| {\tau_{i}^{ + } } \right| = \left| {\tau_{i}^{ - } } \right|$,the saturation model is symmetric, else if $\left| {\tau_{i}^{ + } } \right| \ne \left| {\tau_{i}^{ - } } \right|$ means the actuator has asymmetric saturation.

### MSV model

Neglecting the motions in heave, pitch and roll, the three degrees-of-freedom nonlinear mathematical model of the MSV with disturbances can be considered as Ref.^11^

5 $$ \left\{ \begin{gathered} \dot{x} = u\cos \left( \psi \right) - v\sin \left( \psi \right) \hfill \\ \dot{y} = u\sin \left( \psi \right) + v\cos \left( \psi \right) \hfill \\ \dot{\psi } = r \hfill \\ \end{gathered} \right. $$

where $\left( {x,\,y} \right)$ is the position of the ship,$\psi$ is yaw angle. $u$, $v$, and $r$ are the surge, the sway and the angular velocity of yaw, respectively.

Their derivatives are shown as

6 $$ \left\{ \begin{gathered} \dot{u} = f_{u} (u,v,r) + \frac{1}{{m_{u} }}\left[ {\begin{array}{*{20}l} \tau \hfill \\ \end{array}_{u} + d_{u} } \right] \hfill \\ \dot{\nu } = f_{v} (u,v,r) + \frac{1}{{m_{v} }}d_{v} \hfill \\ \dot{r} = f_{r} (u,v,r) + \frac{1}{{m_{r} }}\left[ {\begin{array}{*{20}l} \tau \hfill \\ \end{array}_{r} + d_{r} } \right] \hfill \\ \end{gathered} \right. $$

7 $$ \left\{ \begin{gathered} f_{u} \left( {u,v,r} \right) = \frac{1}{{m_{u} }}\left( {m_{v} vr - Y_{{\dot{r}}} r^{2} + X_{u} u + X_{u\left| u \right|} \left| u \right|u} \right) \hfill \\ f_{v} \left( {u,v,r} \right) = \frac{1}{{m_{v} }}\left( {Y_{v} v + Y_{|v|v} |v|v + Y_{|r|v} |r|v + Y_{r} r - m_{u} ur\left. { + Y_{|v|r} |v|r + Y_{|r|r} |r|r} \right)} \right. \hfill \\ f_{r} \left( {u,v,r} \right) = \frac{1}{{m_{r} }}\left[ {\left( {m_{u} - m_{v} } \right)uv + Y_{{\dot{r}}} ur + N_{v} v + N_{r} r + N_{|r|v} |r|v + N_{|v|v} |v|v + N_{|v|r} |v|r + N_{|r|r} |r|r} \right] \hfill \\ \end{gathered} \right. $$

where $$\begin{array}{*{20}l} \tau \hfill \\ \end{array}_{u} = \varphi_{u} + \sigma {(}\varphi_{u} {)}$$ and $$\begin{array}{*{20}l} \tau \hfill \\ \end{array}_{r} = \varphi_{r} + \sigma {(}\varphi_{r} {)}$$ are the inputs, $d_{u}$,$d_{u}$ and $d_{u}$ are unknown dynamics and disturbances. $f_{u} \left( {u,v,r} \right)$, $f_{v} \left( {u,v,r} \right)$ and $f_{r} \left( {u,v,r} \right)$ are the terms of high order dynamics.

#### Assumption 1

The reference signal is a desired smooth function $\eta_{r} \left( {x_{r} ,\,y_{r} ,\,\psi_{r} } \right)$ which are bounded and have the bounded first and second times of derivatives $\dot{\eta }_{r} ,\,\ddot{\eta }_{r}$.There exists a positive constant $B_{{0}}$ with such condition that $\left\| {\eta_{r} } \right\|^{2} + \;\left\| {\dot{\eta }_{r} } \right\|^{2} + \left\| {\ddot{\eta }_{r} } \right\|^{2} \le B_{{0}}$.

#### Assumption 2

Assume that the control command $\tau$, the unknown disturbances $d$ and the optimal weight vector $\omega^{*}$ are bounded.

#### Definition 1

(^35^). Given a nonlinear system $\dot{x} = f\left( {x,t} \right),\;x \in {\mathbb{R}}^{n} ,\;t \ge t_{{0}}$, the solution of the above system is semi-globally uniformly ultimately bounded if for any $\Omega_{{0}}$, a compact subset of ${\mathbb{R}}^{n}$ and all $\dot{x}\left( {t_{{0}} } \right) = f\left( {x,t} \right),\;x \in \Omega_{{0}}$, there exists $S > {0}$ and a number $T\left( {S,X\left( {t_{{0}} } \right)} \right)$ such that $\left\| {X\left( t \right)} \right\| \le S$ for all $t \ge t_{{0}} + T$.

#### Lemma 1

(^35^). The condition $\dot{V}\left( x \right) + \lambda_{1} V\left( x \right) + \lambda_{2} V^{l} \left( x \right) \le 0$ is satisfied by the existence of real number $\lambda_{1} > 0,\lambda_{2} > 0,l \in \left( {0,1} \right)$ and an open-loop neighborhood near the origin. Then the origin is stable in finite time, and the stable time is

8 $$ T_{r} = \frac{1}{{\lambda_{1} (1 - l)}}\ln \frac{{\lambda_{1} V^{1 - l} (x) + \lambda_{2} }}{{\lambda_{2} }}. $$

#### Lemma 2

(^36^). For any constant $a > {0}$ and $x \in R$, it satisfies ${0 < }\left| x \right| - x{\text{tanh}}\left( \frac{x}{a} \right) \le 0.2785a$.

## Controller design

In this section, we design the trajectory tracking controller for the MSV model as stated in Section “Preliminaries and problem statements”, all states of the MSV are assume to be measurable. Firstly, define tracking error of underactuated MSV as

9 $$ \left\{ \begin{gathered} x_{e} = x - x^{*} \hfill \\ y_{e} = y - y^{*} \hfill \\ \psi_{e} = \psi - \psi^{*} \hfill \\ \end{gathered} \right. $$

Then let

10 $$ z_{e} = \left[ \begin{gathered} x_{e} \hfill \\ y_{e} \hfill \\ \end{gathered} \right] = \left[ \begin{gathered} x - x^{*} \hfill \\ y - y^{*} \hfill \\ \end{gathered} \right] $$

Thus, derivatives can be obtained as

11 $$ \dot{z}_{e} = ug_{u} \left( \varphi \right) + vg_{v} \left( \varphi \right) - \left( \begin{gathered} \dot{x}^{*} \hfill \\ \dot{y}^{*} \hfill \\ \end{gathered} \right) $$

where $$g_{u} \left( \varphi \right) = \left[ \begin{gathered} \cos \left( \varphi \right) \hfill \\ \sin \left( \varphi \right) \hfill \\ \end{gathered} \right]$$, $$g_{v} \left( \varphi \right) = \left[ \begin{gathered} - \sin \left( \varphi \right) \hfill \\ \, \cos \left( \varphi \right) \hfill \\ \end{gathered} \right]$$.

According to formula (11), the virtual control law is designed as

12 $$ \left\{ \begin{gathered} \alpha = - k_{11} z_{e} - \frac{{k_{12} z_{e} }}{{\sqrt {\left\| {z_{e} } \right\|^{2} + \delta^{2} } }} - vg_{v} \left( \psi \right) + \left( \begin{gathered} \dot{x}^{*} \hfill \\ \dot{y}^{*} \hfill \\ \end{gathered} \right) \hfill \\ \alpha_{r} = - k_{31} \psi_{e} - \frac{{k_{32} \psi_{e} }}{{\sqrt {\left\| {\psi_{e} } \right\|^{2} + \delta^{2} } }} + \dot{\psi }^{*} \hfill \\ \end{gathered} \right. $$

The speed, heading rate and heading angle are expected as

13 $$ \left\{ \begin{gathered} u^{*} = \left\| \alpha \right\| \hfill \\ r^{*} = - k_{31} \psi_{e} - \frac{{k_{32} \psi_{e} }}{{\sqrt {\left\| {\psi_{e} } \right\|^{2} + \delta^{2} } }} + \dot{\psi }^{*} \hfill \\ \psi^{*} = \arctan \left( {\alpha_{y} ,\alpha_{x} } \right) \hfill \\ \end{gathered} \right. $$

Define the following error variables:

14 $$ \left\{ \begin{gathered} u_{e} = u - u^{*} \hfill \\ r_{e} = r - r^{*} \hfill \\ \end{gathered} \right. $$

Take the derivative of Eq. (14), it has

15 $$ \left\{ \begin{gathered} \dot{u}_{e} = f_{u} \left( {u,v,r} \right) + \frac{{1}}{{m_{u} }}\left( {\varphi_{u} + \sigma \left( {\varphi_{u} } \right) + + d_{u} } \right) - \dot{u}^{*} \hfill \\ \dot{r}_{e} = f_{r} \left( {u,v,r} \right) + \frac{{1}}{{m_{r} }}\left( {\varphi_{r} + \sigma \left( {\varphi_{r} } \right) + d_{r} } \right) - \dot{r}^{*} \hfill \\ \end{gathered} \right. $$

where the unknown dynamics are approximated by using RBF neural networks

16 $$ \left\{ \begin{gathered} f_{u} \left( {u,v,r} \right) + \frac{{1}}{{m_{u} }}\sigma {(}\varphi_{u} {)} = W_{u}^{T} \sigma \left( \eta \right) + \varepsilon_{u} \hfill \\ f_{r} \left( {u,v,r} \right) + \frac{{1}}{{m_{r} }}\sigma {(}\varphi_{r} {)} = W_{r}^{T} \sigma \left( \eta \right) + \varepsilon_{r} \hfill \\ \end{gathered} \right. $$

Thus, the following control law is designed

17 $$ \left\{ \begin{gathered} \varphi_{u} = m_{u} \left[ { - k_{21} u_{e} - \frac{{k_{22} u_{e} }}{{\sqrt {\left\| {u_{e} } \right\|^{2} + \varsigma^{2} } }} - \hat{W}_{u}^{T} \sigma \left( \eta \right) + \dot{u}^{*} - {\text{Tanh}} \left( {\frac{{u_{e} }}{{v_{u} }}} \right)\hat{\overline{\delta }}_{u} } \right] \hfill \\ \varphi_{r} = m_{r} \left[ { - k_{41} r_{e} - \frac{{k_{42} r_{e} }}{{\sqrt {\left\| {r_{e} } \right\|^{2} + \varsigma^{2} } }} - \hat{W}_{r}^{T} \sigma \left( \eta \right) - \psi_{e} + \dot{r}^{*} - {\text{Tanh}} (\frac{{r_{e} }}{{v_{r} }})\hat{\overline{\delta }}_{r} } \right] \hfill \\ \end{gathered} \right. $$

where $\delta_{u} = \varepsilon_{u} + \tau_{d,u}$, $\delta_{r} = \varepsilon_{r} + \tau_{d,r}$, $\hat{\overline{\delta }}_{u} ,\hat{\overline{\delta }}_{r}$ are the estimations of the upper bounds.

The adaptive law is designed as follows

18 $$ \left\{ \begin{gathered} \dot{\hat{W}}_{u} = \gamma_{{w_{u} }} {[}u_{e} \sigma (\eta ) - \lambda_{{w_{u} }} \left\| {u_{e} } \right\|\hat{W}_{u} {]} \hfill \\ \dot{\hat{W}}_{r} = \gamma_{{w_{r} }} {[}r_{e} \sigma (\eta ) - \lambda_{{w_{r} }} \left\| {r_{e} } \right\|\hat{W}_{r} {]} \hfill \\ \end{gathered} \right. $$

19 $$ \left\{ \begin{gathered} \dot{\hat{\overline{\delta }}}_{u} { = }\gamma_{{d_{u} }} \,\left( {{\text{Tanh}}\left( {\frac{{u_{e} }}{{\varpi_{u} }}} \right)u_{e} - \lambda_{{d_{u} }} \hat{\overline{\delta }}_{u} } \right) \hfill \\ \dot{\hat{\overline{\delta }}}_{r} { = }\gamma_{{d_{r} }} \left( {{\text{Tanh}}\left( {\frac{{r_{e} }}{{\varpi_{r} }}} \right)r_{e} - \lambda_{{d_{r} }} \hat{\overline{\delta }}_{u} } \right) \hfill \\ \end{gathered} \right. $$

Define errors as

20 $$ \begin{gathered} e_{u} \left( t \right) = \omega_{u} \left( t \right) - \delta_{u} \left( t \right), \, t \in \left[ {t_{k} ,t_{k + 1} } \right) \hfill \\ e_{r} \left( t \right) = \omega_{r} \left( t \right) - \delta_{r} \left( t \right), \, t \in \left[ {t_{k} ,t_{k + 1} } \right) \hfill \\ \end{gathered} $$

Design trigger conditions

21 $$ \left\{ \begin{gathered} \omega_{u} \left( t \right) = \delta_{u} \left( t \right), \, t \in \left[ {t_{k} ,t_{k + 1} } \right) \hfill \\ t_{k + 1} = \inf \left\{ {t \in R\left| {\eta_{u} \left( t \right) + h_{14} \left[ {h_{12} \left| {\omega_{u} \left( t \right)} \right| - h_{13} \left| {e_{u} } \right|} \right] \ge 0} \right.} \right\} \hfill \\ \end{gathered} \right. $$

22 $$ \left\{ \begin{gathered} \omega_{r} \left( t \right) = \delta_{r} \left( t \right), \, t \in \left[ {t_{k} ,t_{k + 1} } \right) \hfill \\ t_{k + 1} = \inf \left\{ {t \in R\left| {\eta_{r} \left( t \right) + h_{24} \left[ {h_{22} \left| {\omega_{r} \left( t \right)} \right| - h_{23} \left| {e_{r} } \right|} \right] \ge 0} \right.} \right\} \hfill \\ \end{gathered} \right. $$

where $\dot{\eta }_{u} \left( t \right) = - h_{11} \eta_{u} \left( t \right) + h_{12} \left| {\omega_{u} \left( t \right)} \right| - h_{13} \left| {e_{u} \left( t \right)} \right|$, $\dot{\eta }_{r} \left( t \right) = - h_{21} \eta_{r} \left( t \right) + h_{22} \left| {\omega_{r} \left( t \right)} \right| - h_{23} \left| {e_{r} \left( t \right)} \right|$, the event-triggered interval is $\left[ {t_{k} ,t_{k + 1} } \right)$, $\omega_{u} \left( t \right) - \delta_{u} \left( t \right) \le l_{u} ,\,\,\omega_{r} \left( t \right) - \delta_{r} \left( t \right) \le l_{r}$.

## Stability proof

The following Lyapunov function is selected for the underactuated ship kinematics.

23 $$ V = \frac{1}{2}z_{e}^{T} z_{e} + \frac{1}{2}\psi_{e}^{2} + \frac{1}{2}u_{e}^{2} + \frac{1}{2}r_{e}^{2} + \frac{1}{{2\gamma_{{d_{u} }} }}\tilde{\overline{\delta }}_{u}^{2} + \frac{1}{{2\gamma_{{d_{r} }} }}\tilde{\overline{\delta }}_{r}^{2} $$

where $$\tilde{\overline{\delta }}_{u} = \overline{\delta }_{u} - \hat{\overline{\delta }}_{u} ,\tilde{\overline{\delta }}_{r} = \overline{\delta }_{r} - \hat{\overline{\delta }}_{r}$$.

Then, the derivation of (23) is obtained as

24 $$ \dot{V} = z_{e}^{T} \dot{z}_{e} + \psi_{e} \dot{\psi }_{e} + u_{e} \dot{u}_{e} + r_{e} \dot{r}_{e} - \tilde{\overline{\delta }}_{u} \gamma_{{_{{d_{u} }} }} \dot{\hat{\overline{\delta }}}_{u} - \tilde{\overline{\delta }}_{r} \gamma_{{d_{r} }} \dot{\hat{\overline{\delta }}}_{r} $$

25 $$ \begin{gathered} z_{e}^{T} \dot{z}_{e} = - z_{e}^{T} k_{11} z_{e} - \frac{{z_{e}^{T} k_{12} z_{e} }}{{\sqrt {\left\| {z_{e} } \right\|^{2} + \varsigma^{2} } }} + z_{e}^{T} \Delta_{e} \hfill \\ \, \le - z_{e}^{T} k_{11} z_{e} - \frac{{z_{e}^{T} k_{12} z_{e} }}{{\sqrt {\left\| {z_{e} } \right\|^{2} + \varsigma^{2} } }} + z_{e}^{T} \Delta_{e}^{*} \hfill \\ \end{gathered} $$

26 $$ \psi_{e} \dot{\psi }_{e} = - k_{31} \psi_{e}^{2} - \frac{{k_{32} \psi_{e}^{2} }}{{\sqrt {\left\| {\psi_{e} } \right\|^{2} + \varsigma^{2} } }} + \psi_{e} r_{e} $$

27 $$ \begin{gathered} z_{e}^{T} \dot{z}_{e} + \psi_{e} \dot{\psi }_{e} = - z_{e}^{T} k_{11} z_{e} - \frac{{z_{e}^{T} k_{12} z_{e} }}{{\sqrt {\left\| {z_{e} } \right\|^{2} + \varsigma^{2} } }} + z_{e}^{T} \Delta_{e} - k_{31} \psi_{e}^{2} - \frac{{k_{32} \psi_{e}^{2} }}{{\sqrt {\left\| {\psi_{e} } \right\|^{2} + \varsigma^{2} } }} + \psi_{e} r_{e} \hfill \\ \, \le - z_{e}^{T} \kappa_{11} z_{e} - \frac{{z_{e}^{T} k_{12} z_{e} }}{{\sqrt {\left\| {z_{e} } \right\|^{2} + \varsigma^{2} } }} + z_{e}^{T} \Delta_{e}^{*} - \kappa_{11} \psi_{e}^{2} - \frac{{k_{32} \psi_{e}^{2} }}{{\sqrt {\left\| {\psi_{e} } \right\|^{2} + \varsigma^{2} } }} + \psi_{e} r_{e} \hfill \\ \end{gathered} $$

where $\kappa_{11} = \min \left\{ {k_{11} ,k_{31} } \right\}$,$\Delta_{e} = ug_{u} \left( \psi \right) - u^{*} g_{u} \left( {\psi^{*} } \right)$.$u_{e} \dot{u}_{e} - \tilde{\overline{\delta }}_{u} \dot{\hat{\delta }}_{u}$ and $r_{e} \dot{r}_{e} - \tilde{\overline{\delta }}_{r} \dot{\hat{\delta }}_{r}$ can be expressed as

28 $$ \begin{gathered} u_{e} \dot{u}_{e} - \tilde{\overline{\delta }}_{u} \gamma_{{d_{u} }}^{ - 1} \dot{\hat{\delta }}_{u} = u_{e} \left[ { - k_{21} r_{e} - \frac{{k_{22} u_{e} }}{{\sqrt {\left\| {u_{e} } \right\|^{2} + \varsigma^{2} } }} - {\text{Tanh}}\left( {\frac{{u_{e} }}{{\varpi_{u} }}} \right)\hat{\overline{\delta }}_{u} + \tilde{W}_{u}^{T} \sigma \left( \eta \right) + \lambda_{u} \left( t \right)l_{u} } \right] \hfill \\ \, - \tilde{\overline{\delta }}_{u} \left[ {{\text{Tanh}}\left( {\frac{{u_{e} }}{{\varpi_{u} }}} \right)u_{e} - \lambda_{{d_{u} }} \hat{\overline{\delta }}_{u} } \right] \hfill \\ \, \le - \left( {k_{21} + 0.5} \right)u_{e}^{2} - \frac{{k_{22} u_{e}^{2} }}{{\sqrt {\left\| {u_{e} } \right\|^{2} + \varsigma^{2} } }} - \frac{{\lambda_{{d_{u} }} }}{2}\tilde{\overline{\delta }}_{u}^{2} + \frac{1}{4}\left\| {\tilde{W}_{u}^{T} \sigma \left( \eta \right)} \right\|^{2} + \frac{1}{2}\left| {\lambda_{u} \left( t \right)l_{u} } \right|^{2} \hfill \\ \, + u_{e} \left[ {\delta_{u} - {\text{Tanh}}\left( {\frac{{u_{e} }}{{\varpi_{u} }}} \right)\overline{\delta }_{u} } \right] + \frac{{\lambda_{{d_{u} }} }}{2}\overline{\delta }_{u}^{2} \hfill \\ \end{gathered} $$

29 $$ \begin{gathered} r_{e} \dot{r}_{e} - \tilde{\overline{\delta }}_{r} \gamma_{{d_{r} }}^{ - 1} \dot{\hat{\delta }}_{r} = r_{e} \left[ { - k_{41} r_{e} - \frac{{k_{42} r_{e} }}{{\sqrt {\left\| {r_{e} } \right\|^{2} + \varsigma^{2} } }} - \psi_{e} - {\text{Tanh}}\left( {\frac{{r_{e} }}{{\varpi_{r} }}} \right)\hat{\overline{\delta }}_{r} + \tilde{W}_{r}^{T} \sigma \left( \eta \right) + \lambda_{r} \left( t \right)l_{r} } \right] \hfill \\ \, - \tilde{\overline{\delta }}_{u} \left[ {{\text{Tanh}}\left( {\frac{{r_{e} }}{{\varpi_{r} }}} \right)r_{e} - \lambda_{{d_{r} }} \hat{\overline{\delta }}_{r} } \right] \hfill \\ \, \le - \left( {k_{41} + 0.5} \right)r_{e}^{2} - \frac{{k_{42} r_{e}^{2} }}{{\sqrt {\left\| {r_{e} } \right\|^{2} + \varsigma^{2} } }} - r_{e} \psi_{e} - \frac{{\lambda_{{d_{r} }} }}{2}\tilde{\overline{\delta }}_{r}^{2} + \frac{1}{4}\left\| {\tilde{W}_{r}^{T} \sigma \left( \eta \right)} \right\|^{2} \hfill \\ \, + r_{e} \left[ {\delta_{r} - {\text{Tanh}}\left( {\frac{{r_{e} }}{{\varpi_{r} }}} \right)\overline{\delta }_{r} } \right] + \frac{{\lambda_{{d_{r} }} }}{2}\overline{\delta }_{r}^{2} + \frac{1}{2}\left| {\lambda_{r} \left( t \right)l_{r} } \right|^{2} \hfill \\ \end{gathered} $$

According to lemma $0 < \left| x \right| - x{\text{tanh(}}\frac{x}{a}{)} \le {0}{\text{.2785a}}$, obtain

30 $$ \left\{ \begin{gathered} u_{e} \,\left[ {\delta_{u} - {\text{Tanh}}\left( {\frac{{u_{e} }}{{\varpi_{u} }}} \right)\overline{\delta }_{u} } \right] \le 0.2785\varpi_{u} \overline{\delta }_{u} \hfill \\ r_{e} \,\left[ {\delta_{r} - {\text{Tanh}}\left( {\frac{{r_{e} }}{{\varpi_{r} }}} \right)\overline{\delta }_{r} } \right] \le 0.2785\varpi_{r} \overline{\delta }_{r} \hfill \\ \end{gathered} \right. $$

Then, the following inequation is derived from formula (25), (26), (27) and (28),

31 $$ \begin{gathered} u_{e} \dot{u}_{e} - \tilde{\overline{\delta }}_{u} \gamma_{{d_{u} }}^{ - 1} \dot{\hat{\delta }}_{u} \le - \left( {k_{21} + 0.5} \right)u_{e}^{2} - \frac{{k_{22} u_{e}^{2} }}{{\sqrt {\left\| {r_{e} } \right\|^{2} + \varsigma^{2} } }} - \frac{{\lambda_{{d_{u} }} }}{2}\tilde{\overline{\delta }}_{u}^{2} + \frac{{\lambda_{{d_{u} }} }}{2}\overline{\delta }_{u}^{2} \hfill \\ \, + 0.2785\varpi_{u} \overline{\delta }_{u} + \frac{1}{4}\Theta_{u}^{2} + \frac{1}{2}\left| {\lambda_{u} \left( t \right)l_{u} } \right| \hfill \\ \end{gathered} $$

32 $$ \begin{gathered} r_{e} \dot{r}_{e} - \tilde{\overline{\delta }}_{r} \gamma_{{d_{r} }}^{ - 1} \dot{\hat{\delta }}_{r} \le - \left( {k_{41} + 0.5} \right)r_{e}^{2} - \frac{{k_{42} r_{e}^{2} }}{{\sqrt {\left\| {r_{e} } \right\|^{2} + \varsigma^{2} } }} - r_{e} \psi_{e} - \frac{{\lambda_{{w_{r} }} }}{2}\tilde{\overline{\delta }}_{r}^{2} \hfill \\ \, + \frac{{\lambda_{{w_{r} }} }}{2}\overline{\delta }_{r}^{2} + 0.2785\varpi_{r} \delta_{r} + \frac{1}{4}\Theta_{r}^{2} + \frac{1}{2}\left| {\lambda_{r} \left( t \right)l_{r} } \right|^{2} \hfill \\ \end{gathered} $$

Substitute formula (25), (26), (27), (28), (29), (31) and (32) into formula (23)

33 $$ \begin{gathered} \dot{V} \le - z_{e}^{T} \kappa_{11} z_{e} - \frac{{z_{e}^{T} k_{12} z_{e} }}{{\sqrt {\left\| {z_{e} } \right\|^{2} + \varsigma^{2} } }} + z_{e}^{T} \Delta_{e} - \kappa_{11} \psi_{e}^{2} - \frac{{k_{32} \psi_{e}^{2} }}{{\sqrt {\left\| {\psi_{e} } \right\|^{2} + \varsigma^{2} } }} - k_{21} u_{e}^{2} - \frac{{k_{22} u_{e}^{2} }}{{\sqrt {\left\| {u_{e} } \right\|^{2} + \varsigma^{2} } }} - \frac{{\lambda_{{d_{u} }} }}{2}\tilde{\overline{\delta }}_{u}^{2} \hfill \\ + \frac{{\lambda_{{d_{u} }} }}{2}\overline{\delta }_{u}^{2} + 0.2785\varpi_{u} \overline{\delta }_{u} - k_{41} r_{e}^{2} - \frac{{k_{42} r_{e}^{2} }}{{\sqrt {\left\| {r_{e} } \right\|^{2} + \varsigma^{2} } }} - \frac{{\lambda_{{d_{r} }} }}{2}\tilde{\overline{\delta }}_{r}^{2} + \frac{{\lambda_{{d_{r} }} }}{2}\overline{\delta }_{r}^{2} + 0.2785\varpi_{r} \overline{\delta }_{r} + \Lambda \hfill \\ \, \le - z_{e}^{T} \left( {\kappa_{11} { + }0.1} \right)z_{e} - \kappa_{12} \left\| {z_{e} } \right\| - \kappa_{11} \psi_{e}^{2} - \kappa_{12} \left\| {\psi_{e} } \right\| - \kappa_{21} u_{e}^{2} - \kappa_{22} \left\| {u_{e} } \right\| - \frac{{\lambda_{{d_{u} }} }}{2}\tilde{\overline{\delta }}^{2}_{u} + \frac{{\lambda_{{d_{r} }} }}{2}\overline{\delta }_{u}^{2} \hfill \\ + 0.2785\varpi_{u} \overline{\delta }_{u} - \kappa_{21} r_{e}^{2} - \kappa_{22} \left\| {r_{e} } \right\| - \frac{{\lambda_{{d_{r} }} }}{2}\tilde{\overline{\delta }}^{2}_{r} + \frac{{\lambda_{{d_{r} }} }}{2}\overline{\delta }_{r}^{2} + 0.2785\varpi_{r} \overline{\delta }_{r} + \varsigma {[}\kappa_{12} + \kappa_{22} {] + }\Lambda \hfill \\ \end{gathered} $$

where $$\Lambda = \frac{1}{4}\Theta_{u}^{2} + \frac{1}{4}\Theta_{r}^{2} { + }\frac{5}{2}\left\| {\Delta_{e} } \right\|^{2}$$,$$\kappa_{12} = \min \left\{ {k_{12} ,k_{32} } \right\}$$,$$\kappa_{21} = \min \left\{ {k_{21} + 0.5,k_{41} + 0.5} \right\}$$,$$\kappa_{22} = \min \left\{ {k_{22} ,k_{42} } \right\}$$.

By young's inequality, we get

34 $$ \left\{ \begin{gathered} \frac{{\lambda_{{d_{u} }} }}{4}\left\| {\tilde{\overline{\delta }}_{u} } \right\| \le \frac{{\lambda_{{d_{u} }} }}{4}\left\| {\tilde{\overline{\delta }}_{u} } \right\|^{2} + \frac{{\lambda_{{d_{u} }} }}{16} \hfill \\ \frac{{\lambda_{{d_{r} }} }}{4}\left\| {\tilde{\overline{\delta }}_{r} } \right\| \le \frac{{\lambda_{{d_{r} }} }}{4}\left\| {\tilde{\overline{\delta }}_{r} } \right\|^{2} + \frac{{\lambda_{{d_{r} }} }}{16} \hfill \\ \end{gathered} \right. $$

Then obtain

35 $$ \begin{gathered} \dot{V} \le - z_{e}^{T} \left( {\kappa_{11} { + }0.1} \right)z_{e} - \kappa_{12} \left\| {z_{e} } \right\| - \kappa_{21} u_{e}^{2} - \kappa_{11} \psi_{e}^{2} - \kappa_{12} \left\| {\psi_{e} } \right\| - \kappa_{21} r_{e}^{2} - \kappa_{22} \left\| {u_{e} } \right\| - \frac{{\lambda_{{d_{u} }} }}{4}\tilde{\overline{\delta }}^{2}_{u} - \frac{{\lambda_{{d_{u} }} }}{4}\left\| {\tilde{\overline{\delta }}_{u} } \right\| + \frac{{\lambda_{{d_{u} }} }}{2}\overline{\delta }_{u}^{2} \hfill \\ + 0.2785\varpi_{u} \overline{\delta }_{u} - \kappa_{22} \left\| {r_{e} } \right\| - \frac{{\lambda_{{d_{r} }} }}{4}\tilde{\overline{\delta }}^{2}_{r} - \frac{{\lambda_{{d_{r} }} }}{4}\left\| {\tilde{\overline{\delta }}_{r} } \right\| + \frac{{\lambda_{{d_{r} }} }}{2}\overline{\delta }_{r}^{2} + 0.2785\varpi_{r} \overline{\delta }_{r} + \frac{1}{16}\left( {\lambda_{{d_{u} }} + \lambda_{{d_{r} }} } \right) + \varsigma {[}\kappa_{12} + \kappa_{22} {] + }\Lambda \hfill \\ \, \le - \frac{{\rho_{1} }}{2}\left( {z_{e}^{T} z_{e} + u_{e}^{2} + \tilde{\overline{\delta }}_{u}^{T} \lambda_{u}^{ - 1} \tilde{\overline{\delta }}_{u} + \psi_{e}^{2} + r_{e}^{2} + \tilde{\overline{\delta }}_{r}^{T} \lambda_{r}^{ - 1} \tilde{\overline{\delta }}_{r} } \right) \hfill \\ - \rho_{2} \left[ {\left( {\frac{1}{2}z_{e}^{T} z_{e} } \right)^{\frac{1}{2}} + \left( {\frac{1}{2}u_{e}^{2} } \right)^{\frac{1}{2}} + {(}\frac{1}{{2\lambda_{u} }}\tilde{\overline{\delta }}_{u}^{T} \tilde{\overline{\delta }}_{u} {) + }\left( {\frac{1}{2}\psi_{e}^{2} } \right)^{\frac{1}{2}} + \left( {\frac{1}{2}r_{e}^{2} } \right)^{\frac{1}{2}} + {(}\frac{1}{{2\lambda_{r} }}\tilde{\overline{\delta }}_{r}^{T} \tilde{\overline{\delta }}_{r} {)}} \right]{ + }\theta \hfill \\ \, \le - \rho_{1} V - \rho_{2} V^{{\tfrac{1}{2}}} + \theta \hfill \\ \end{gathered} $$

36 $$ \rho_{1} = \min \left\{ {2\kappa_{11} ,2\kappa_{21} ,\frac{{\kappa_{\lambda } }}{2}\kappa_{\gamma } } \right\} $$

37 $$ \rho_{2} = 2^{\frac{1}{2}} \min \left\{ {\kappa_{12} ,\kappa_{22} ,\frac{{\kappa_{\lambda } }}{4}\lambda_{m}^{1/2} \left( {\kappa_{\gamma } } \right)} \right\} $$

38 $$ \kappa_{\lambda } = \min \left\{ {\lambda_{{d_{u} }} ,\lambda_{{d_{r} }} } \right\},\kappa_{{_{\gamma } }} = \min \left\{ {\gamma_{{d_{u} }} ,\gamma_{{d_{r} }} } \right\} $$

39 $$ \theta = \frac{{\kappa_{\lambda } }}{2}\left( {\overline{\delta }_{u}^{2} + \overline{\delta }_{r}^{2} } \right) + 0.2785\left( {\varpi_{u} \overline{\delta }_{u} + \varpi_{r} \overline{\delta }_{r} } \right) + \frac{{\kappa_{\lambda } }}{16}{\kern 1pt} + \varsigma {[}\kappa_{12} + \kappa_{22} {] + }\Lambda $$

We can get a result according to formula (35)

40 $$ \dot{V} \le - \iota \rho_{1} V - \left( {1 - \iota } \right)\rho_{1} V - \rho_{2} V^{{\tfrac{1}{2}}} + \theta $$

where $$\iota = \min \left\{ {\iota_{{1}} ,\iota_{{2}} } \right\}$$, $${0} < \iota < {1}$$.

According to formula (28), if $V > \theta /\iota \rho_{1}$, we have

41 $$ \dot{V} \le - \left( {1 - \iota } \right)\rho_{1} V - \rho_{2} V^{{\tfrac{1}{2}}} $$

According to Lemma 1, the system will stabilize to the region in finite time $\Omega_{V} = \left\{ {V:V \le \theta /\imath \rho_{1} } \right\}$ and the stability time is

42 $$ T \le \frac{4}{{\left( {1 - t} \right)\rho_{1} }}\ln \left[ {\frac{{\left( {1 - t} \right)\rho_{1} V^{1/2} \left( 0 \right) + \rho_{2} }}{{\rho_{2} }}} \right] $$

where $V\left( {0} \right)$ is the initial value of $V$.

For formula (42), it guarantees the system converge in finite time $\forall t \ge T$, that has

43 $$ \left\{ \begin{gathered} \frac{d}{dt}|e_{u} | = \frac{d}{dt}\left( {e_{u} .e_{u} } \right)^{\frac{1}{2}} = {\text{sign}} (e_{u} )\dot{e}_{u} \le |\dot{\delta }_{u} (t)| \hfill \\ \frac{d}{dt}|e_{r} | = \frac{d}{dt}\left( {e_{r} .e_{r} } \right)^{\frac{1}{2}} = {\text{sign}} (e_{r} )\dot{e}_{r} \le |\dot{\delta }_{r} (t)| \hfill \\ \end{gathered} \right. $$

Further, it can be obtained smooth continuous differentiable functions of $\delta_{u}$ and $\delta_{r}$.

44 $$ \left\{ {\begin{array}{*{20}l} {\dot{\delta }_{u} = m_{u} \{ - k_{21} \dot{u}_{e} - \frac{{k_{22} \dot{u}_{e} }}{{\sqrt {\left\| {u_{e} } \right\|^{2} + \varsigma_{u}^{2} } }} - \dot{\hat{W}}_{u}^{T} \sigma \left( \eta \right) + \ddot{u}^{*} - \frac{1}{{\cosh^{2} }}\left( {\frac{{u_{e} }}{{\varpi_{u} }}} \right)\dot{u}_{e} \hat{\overline{\delta }}_{u} - {\text{Tanh}} \left( {\frac{{u_{e} }}{{\varpi_{u} }}} \right)\dot{\hat{\overline{\delta }}}_{u} \} } \hfill \\ {\dot{\delta }_{r} = m_{r} \{ - k_{41} \dot{r}_{e} - \frac{{k_{42} \dot{r}_{e} }}{{\sqrt {\left\| {e_{r} } \right\|^{2} + \varsigma_{r}^{2} } }} - \dot{\hat{W}}_{r}^{T} \sigma \left( \eta \right) + \ddot{r}^{*} - \frac{1}{{\cosh^{2} }}\left( {\frac{{r_{e} }}{{\varpi_{r} }}} \right)\dot{r}_{e} \hat{\overline{\delta }}_{r} - {\text{Tanh}} \left( {\frac{{r_{e} }}{{\varpi_{r} }}} \right)\dot{\hat{\overline{\delta }}}_{r} \} } \hfill \\ \end{array} } \right. $$

Since all variables in $\dot{\delta }_{u}$ and $\dot{\delta }_{r}$ are globally bounded, there exists constants $\vartheta_{u} > 0$,$\vartheta_{r} > 0$, such that the condition $\left| {\dot{\omega }_{u} } \right| < \vartheta_{u} ,\;\left| {\dot{\omega }_{r} } \right| < \vartheta_{r}$ are satisfied. When $t = t_{k}$,$e_{u} \left( {t_{k} } \right)$ and $e_{r} \left( {t_{k} } \right)$ are 0,$\mathop {\lim }\limits_{t \to \infty } e_{u} \left( t \right) = \vartheta_{u}$,$\mathop {\lim }\limits_{t \to \infty } e_{r} \left( t \right) = \vartheta_{r}$. Therefore, there is time $t^{*}$ interval satisfaction $t^{*} \ge l_{i} /\vartheta_{i}$,$i = u,r$, so zeno behavior does not occur.

## Simulation

**Figure 1:**
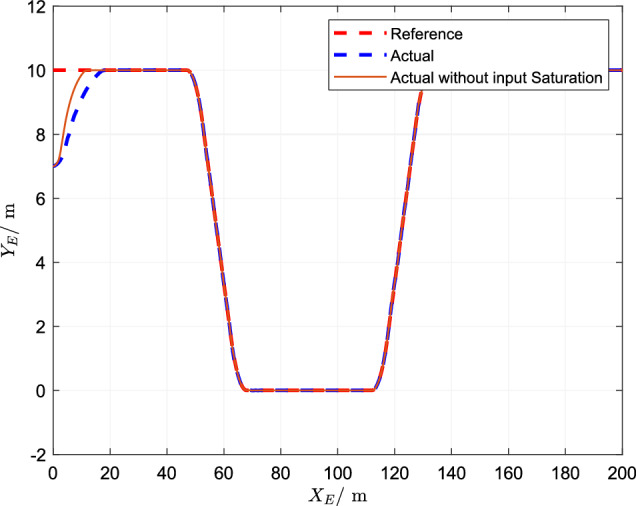
Trajectory of reference tracking.

**Figure 2:**
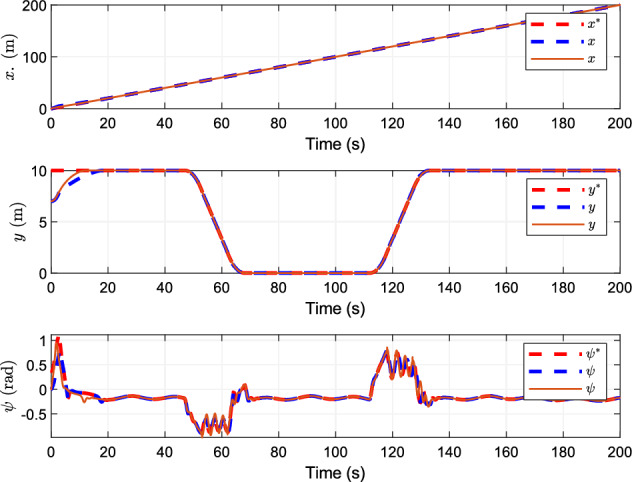
Tracking curves of position and heading.

**Figure 3:**
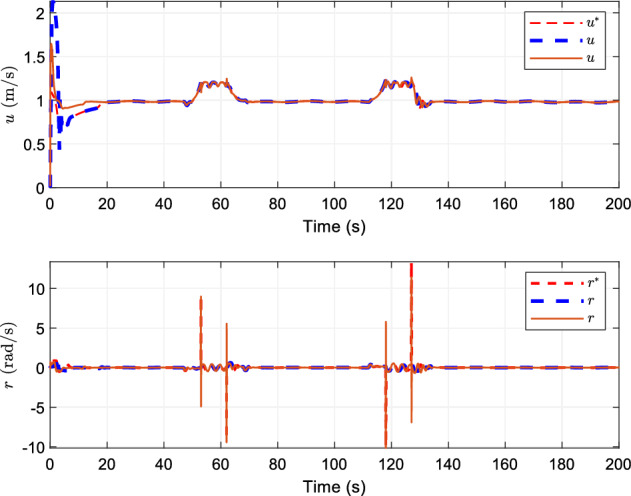
Tracking curves of surge velocity and yaw angle velocity.

**Figure 4:**
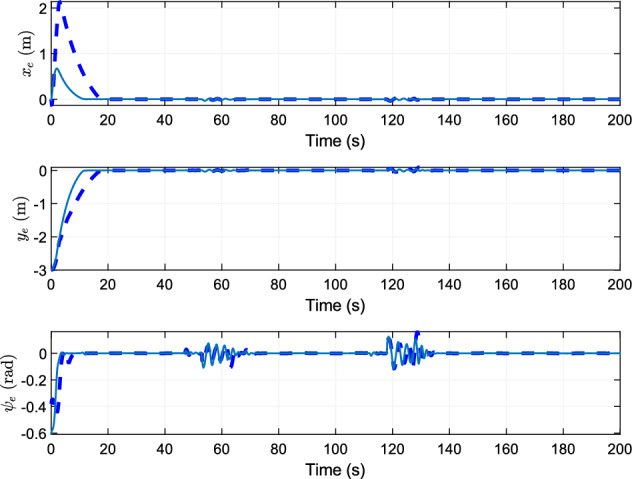
Tracking errors of reference trajectory.

**Figure 5:**
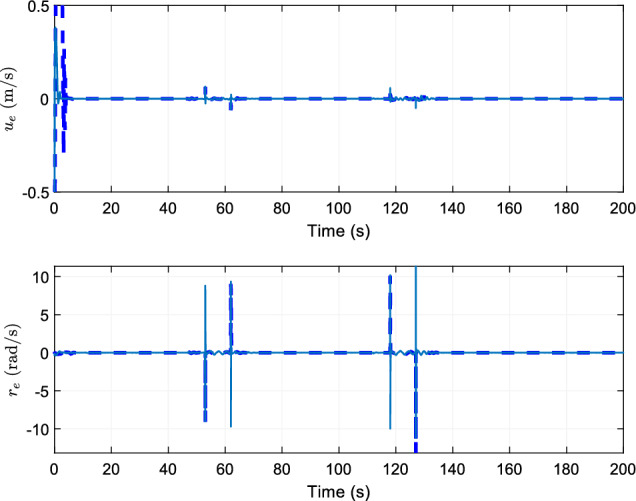
Tracking errors of ship velocity.

**Figure 6:**
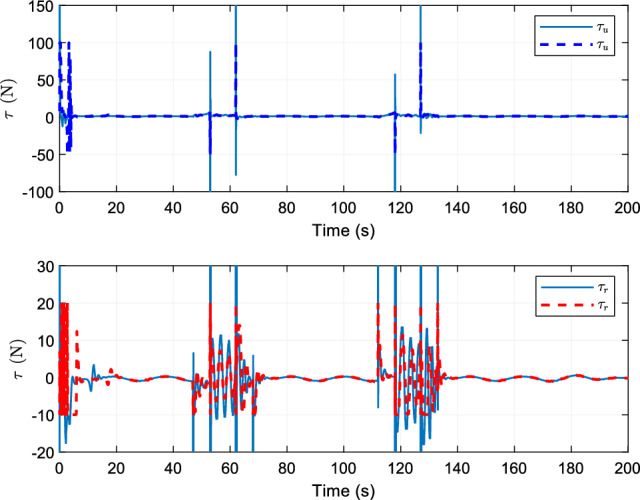
Curves of control system inputs $\varphi_{u}$ and $\varphi_{r}$.

**Figure 7:**
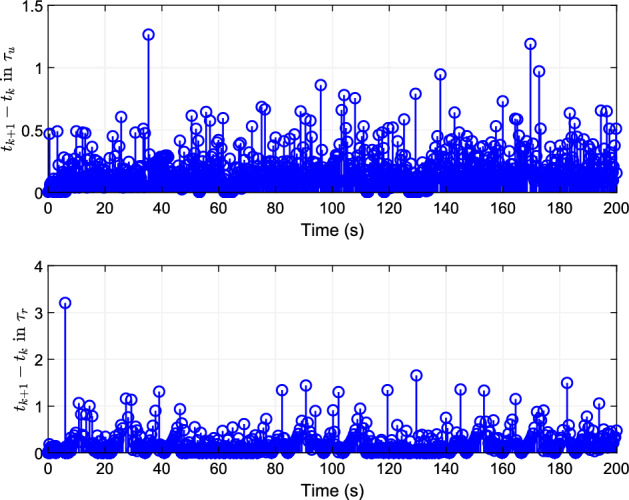
Event-trigger interval of the inputs $\varphi_{u}$ and $\varphi_{r}$.

In the simulation, the length of ship is $L = 1.255m$,$M = 23.8kg$ and other parameters are referred to paper^37^. The designed parameters are set as $k_{11} = 0.1$, $k_{12} = 0.1$, $k_{21} = 0.2$, $k_{22} = 0.1$, $k_{31} = 0.2$, $k_{32} = 0.05$, $k_{41} = 0.4$, $k_{42} = 0.05$.The simulation results are shown in Figs. 1, 2, 3, 4, 5, 6 and 7.

The simulations have been implemented and results of the method adopted in this paper have been shown in the figures using dashed lines.The comparison is provided for the proof of validity. The method without considering asymmetric input saturation is taken as comparison that displayed in solid line mode.

Figure 1 shows the tracking effect of the actual and expected trajectories of the control scheme, and the tracking effect of the control scheme designed in this study is good. Both control schemes can make the actual cures track the reference, and meet the control requirements of the system. However, the finite-time trajectory tracking control scheme designed in this paper considers the existence of asymmetric input saturation when the fault occurs, and still achieves high tracking accuracy.

Figure 2 shows the tracking of the ship's actual position and actual heading angle. Both control schemes realize the tracking of reference position and reference heading. The control scheme designed in this paper shows good robustness in the tracking process of position and heading angle, and has high tracking accuracy for the desired position and heading angle.

Figure 3 shows the speed tracking of the ship, indicating that this research scheme can track the desired speed in a limited time. The changes of the actual speed of the ship and the tracking effect of the reference speed of the two control schemes tend to be similar with time, and the tracking of the reference speed is achieved.

The change of posture error with time and the change of heading error with time are displayed in Fig. 4. Even there is input saturation, the error at the beginning is limited in a small range. Finally the convergence rate of tracking error is almost the same.

Figure 5 shows the comparison effect of tracking errors of ship velocity. The upper and lower bounds of the errors of the two control schemes are small, which shows the effectiveness of the control scheme designed in this paper.

Figure 6 shows the comparison curves of the control inputs. The oscillation amplitude of the control inputs in this study are limited by asymmetric input saturation $- {100} \le \varphi_{u} \le {200}$, $- {10} \le \varphi_{r} \le {20}$. In addition, by analyzing the tracking situation of the reference trajectory of the control scheme in this study, the finite-time trajectory tracking control scheme proposed in this study has strong robustness.

The introduction of event triggering mechanism effectively reduces the update times of controller as show in Fig. 7. By reconstructing the dynamic uncertainty of the ship through the neural networks, the problem of finite time trajectory tracking control is solved considering asymmetric saturation. The tracking effect of the system is guaranteed, and the introduction of the event triggering mechanism effectively saves the communication resources.

## Conclusion

A finite-time trajectory tracking control scheme based on adaptive neural networks with minimum learning parameters and asymmetric input saturation is proposed for underactuated surface ships affected by dynamic uncertainties and external unknown disturbances. The unknown dynamic uncertainty of the ship is approximated by neural network, and the computational complexity is reduced by combining the minimum learning parameter, and the controller structure is simplified. Then, an adaptive law is designed to approximate the upper bound of the composite disturbance to solve the asymmetric input saturation limit problem. Finally, the simulation results show that the proposed control scheme can make all signals in the closed-loop trajectory tracking system bounded, and ensure that the actual trajectory of the ship can track the desired trajectory in finite time. The control scheme designed in this study has good performance and is more suitable for application in engineering practice (The framework of the adaptive finite time trajectory tracking control method, and more simulation results are provided in the Supplementary Figures).

## Supplementary Information

Supplementary Figures.

## Data Availability

All data generated or analysed during this study are included in this published article [and its supplementary information files]. Besides, the datasets used and/or analysed during the current study are available from the corresponding author on reasonable request.
