# Supplementary material for: Event trigger based adaptive neural trajectory tracking finite time control for underactuated unmanned marine surface vessels with asymmetric input saturation
Source: Sci Rep. 2023 Jun 22;13:10126. doi: 10.1038/s41598-023-37331-6 (PMC10287687; doi:10.1038/s41598-023-37331-6)
Supplement: Supplementary file 1 — Supplementary Figures. [file 41598_2023_37331_MOESM1_ESM.docx]

In Figure S1, an adaptive finite time trajectory tracking control method is designed for underactuated unmanned marine surface vessels (MSVs) by using neural networks to approximate system uncertainties, and the simulation results are shown in Figure S2. The proposed adaptive controller is developed by combining event-triggered control (ETC) and finite-time convergence (FTC) techniques considering input asymmetrical saturation. The adaptive laws of controller is shown in Figure S3.

Figure S1. The design of trajectory tracking controller


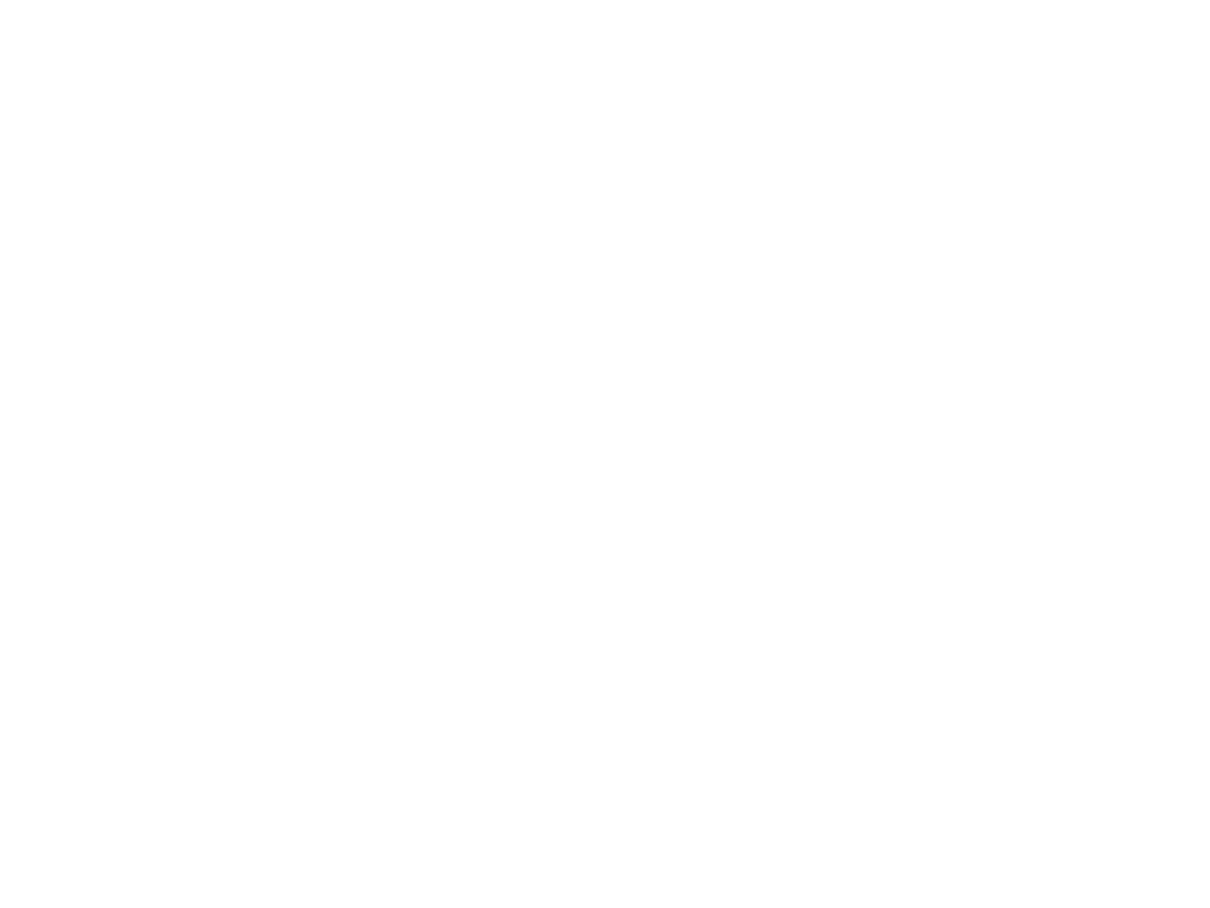


Figure S2. The Neural Networks approximation of uncertainties


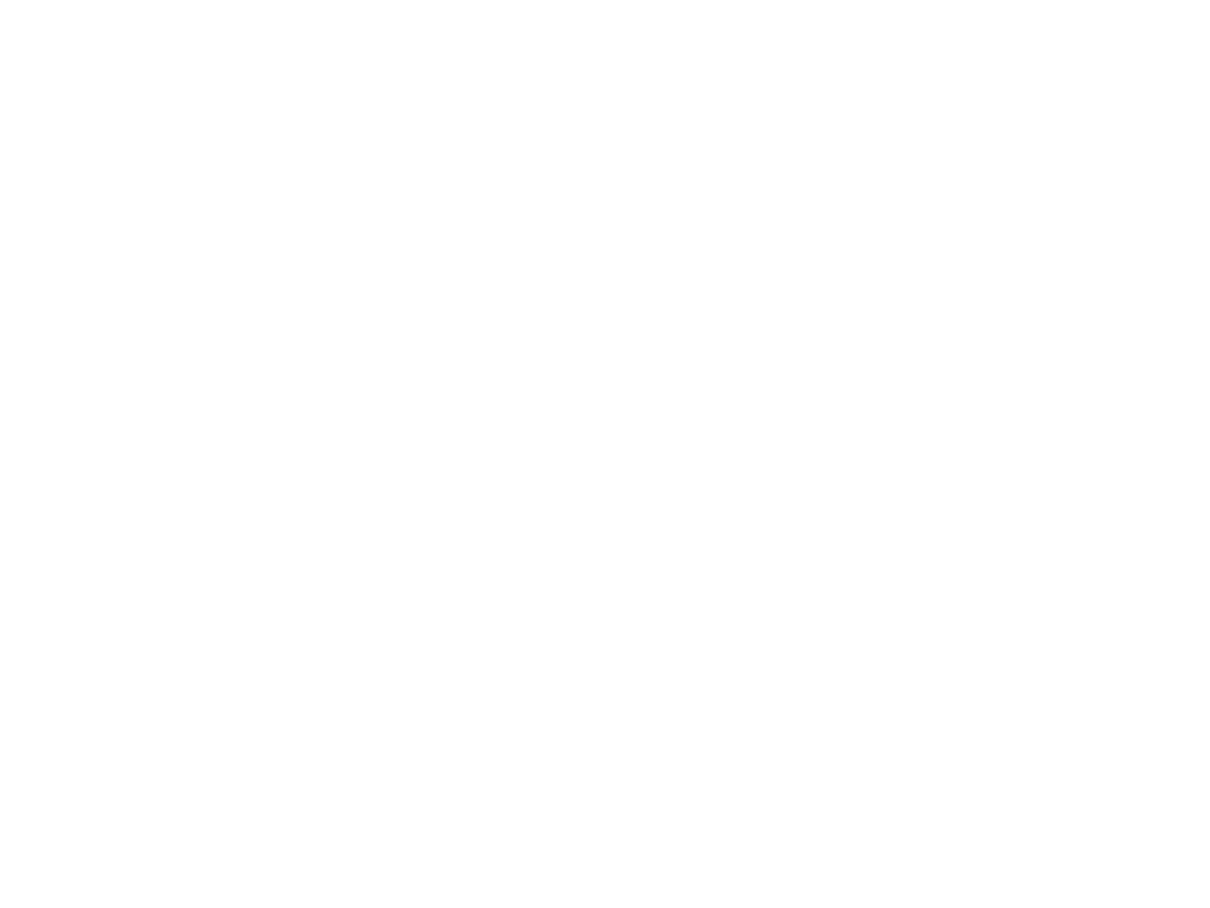


Figure S3. The adaptive laws of controller
